# The effectiveness of artificial intelligence-based automated grading and training system in education of manual detection of diabetic retinopathy

**DOI:** 10.3389/fpubh.2022.1025271

**Published:** 2022-11-07

**Authors:** Xu Qian, Han Jingying, Song Xian, Zhao Yuqing, Wu Lili, Chu Baorui, Guo Wei, Zheng Yefeng, Zhang Qiang, Chu Chunyan, Bian Cheng, Ma Kai, Qu Yi

**Affiliations:** ^1^Department of Geriatrics, Qilu Hospital of Shandong University, Jinan, China; ^2^Key Laboratory of Cardiovascular Proteomics of Shandong Province, Jinan, China; ^3^Jinan Clinical Research Center for Geriatric Medicine (202132001), Jinan, China; ^4^School of Basic Medical Sciences, Shandong University, Jinan, China; ^5^Lunan Eye Hospital, Linyi, China; ^6^Tencent Healthcare, Shenzhen, China

**Keywords:** medical image education, artificial intelligence, diabetic retinopathy, medical students, diagnosis

## Abstract

**Background:**

The purpose of this study is to develop an artificial intelligence (AI)-based automated diabetic retinopathy (DR) grading and training system from a real-world diabetic dataset of China, and in particular, to investigate its effectiveness as a learning tool of DR manual grading for medical students.

**Methods:**

We developed an automated DR grading and training system equipped with an AI-driven diagnosis algorithm to highlight highly prognostic related regions in the input image. Less experienced prospective physicians received pre- and post-training tests by the AI diagnosis platform. Then, changes in the diagnostic accuracy of the participants were evaluated.

**Results:**

We randomly selected 8,063 cases diagnosed with DR and 7,925 with non-DR fundus images from type 2 diabetes patients. The automated DR grading system we developed achieved accuracy, sensitivity/specificity, and AUC values of 0.965, 0.965/0.966, and 0.980 for moderate or worse DR (95 percent CI: 0.976–0.984). When the graders received assistance from the output of the AI system, the metrics were enhanced in varying degrees. The automated DR grading system helped to improve the accuracy of human graders, i.e., junior residents and medical students, from 0.947 and 0.915 to 0.978 and 0.954, respectively.

**Conclusion:**

The AI-based systemdemonstrated high diagnostic accuracy for the detection of DR on fundus images from real-world diabetics, and could be utilized as a training aid system for trainees lacking formal instruction on DR management.

## Introduction

Diabetic Retinopathy (DR) is the leading cause of blindness and visual impairment in the working-age population worldwide (1). Numerous studies have shown that early detection and timely treatment of DR could prevent severe vision loss in more than 90% of diabetics (2, 3). However, due to a severe shortage of retinal specialists, a large proportion of patients in underdeveloped countries were unable to receive annual eye examinations recommended by the protocol (4, 5). In the face of a rapidly rising global diabetes incidence (6), a new approach to diabetes management is urgently needed. It has been confirmed that after receiving training in fundus photographic reading, non-ophthalmologists were highly sensitive as ophthalmologists in detecting DR (7). The training for non-ophthalmological readers seems to be an important step toward their integration into diabetic eye screening.

Accurate clinical staging of DR is a proven prerequisite for choosing the most appropriate personalized treatment. The Early Treatment Diabetic Retinopathy Study (ETDRS) based on color fundus photography is now the gold standard of DR grading (8). Nevertheless, the training procedure of image identification is of great implementation complexity because of individual variations of real-life cases encountered. In order to acquire skills to establish diagnosis in daily clinical practice, the trainees need to learn from a considerable number of images to extract image features. But training opportunities might be compressed due to limitations of resources, staff and finance (9). Furthermore, even highly qualified instructors might be subjective as well as have intra- and inter-reader diagnostic variations (10). Traditional ophthalmology courses often fail to provide a fairly large number of standardized cases for training purposes.

In recent years, artificial intelligence (AI) has shown obvious advantages in diagnosis and prediction of major eye diseases particularly those involving image analysis (11–13). Recent advances in automated retinal image screening systems using AI have demonstrated that specialist-level accuracy was can be achieved inDR assessment (10, 14). The implementations of big data and AI technologies in educational environments have also demonstrated significant potential for enhancing the efficiency of instruction (15). The essential information extracted from big data can help to shorten training periods and improve the learning curve of students. However, AI' s potential as an examination system and/or a robot teacher offering personalized education for medical students and traineesrequires further evaluation.

In this study, we developed an AI-based automated DR grading system equipped with an AI-driven diagnosis algorithm, and validated its role as an instructional and learning tool in training non-ophthalmic physicians in DR manual grading.

## Materials and methods

### The AI-based automated DR grading and training system

The study protocol was approved by the institutional review board of Qilu Hospital of Shandong University (QLHSDU) and conducted in accordance with the Declaration of Helsinki.

### Dataset

78,000 anonymized color fundus images were primarily collected from consecutive patients with diabetes over 40 years old in the diabetes clinic of QLHSDU from January 1st, 2016 to January 14th, 2019. The mean age was 60.82 years (SD 11.34), and 58.44% of the participants were male. Macula-centered fundus images were captured using a Canon CR-2 fundus camera (45° field-of-view) with JPEG compression format (resolution in 18 megapixels). Participants' informed consents were exempted by the institutional review board of QLHSDU because the study was retrospective in nature that used completely anonymized data.

All the collected images were preprocessed by an image quality filter and reviewed by three experienced senior ophthalmologists. Images with severe blur, under-exposure, over exposure or severe cataract, out of focus, and fractional images without optic disc were graded as “poor quality”, as it was impossible to make reasonable diagnosis of DR. Among all the 78,000 images, 19,245 (24.67%) were excluded due to image quality issues, leaving 58,755 with a conclusive DR severity grading in total. 8,063 cases of all the obtained 58,755 images were diagnosed as DR, so we randomly selected 7,925 non-DR fundus images from the remaining dataset in order to balance the data distribution and avoid data overfitting. The DR images were classified into four categories according to the International Clinical Diabetic Retinopathy (ICDR) severity scale (16), and each category was randomly chosen at a ratio of 4:1 to divide the images into a training set and a validation set, to guarantee that there was a similar distribution of data between the training set and the validation set. Of the total 15,988 images, 13,222 images were randomly assigned to the training dataset and the remaining 2,766 images were held for validation.

### Algorithm development

In this study, the underlying AI algorithm of the automated DR grading system was developed by Tencent Healthcare, where deep convolutional neural networks were initially pre-trained on a large volume of fundus images collected from several Chinese hospitals for the 5-stage DR classification task according to the ICDR severity scale. The network models were further fine-tuned with the collected real-world training dataset in this study to accommodate data and annotation variations.

The AI framework consisted of a standard ResNet-50 image classification network and an auxiliary graph convolutional network that integrated the prior class-dependency into the classification task. The prior class-dependency was represented by an adjacency matrix to reflect the correlations of adjacent DR stages. And the values of the adjacency matrix were updated simultaneously within the network training process. The learned prior information was used as residual information in the inference stage to re-rank the original results of the classification network and could potentially boost the performance of the algorithm. More details of the network design were introduced in the previous work (17).

For both training and validation datasets, we cropped the images to the size of 512 × 512 and applied the standard normalization to uniform the pixel values to the range (−1, 1). The Stochastic Gradient Descent (SGD) was utilized as the optimizer and the learning rate was set to 0.0001. Augmentation of the data including random scaling, rotation, horizontal flip, and vertical flip was involved to enlarge the size of the training set.

The network generated DR-stage probabilities for each input image, including none, mild, moderate, severe, and proliferative DR. The category with the highest probability value served as the network prediction. We noted that the annotation used a different fundus range from the ICDR scale, which was susceptible to misclassification in moderate DR and severe DR images. As a result, the modified 4-stage DR classification, including Non-DR, Mild DR, Moderate and Severe DR, and PDR, was implemented to prevent underestimating the prevalence of diabetic retinopathy. While the network models were trained for the multi-stage DR classification, we also analyzed the model performance on a binary classification task, i.e., referable DR vs. non-referable DR, where referable DR was defined as moderate DR or worse.

**Figure 1:**
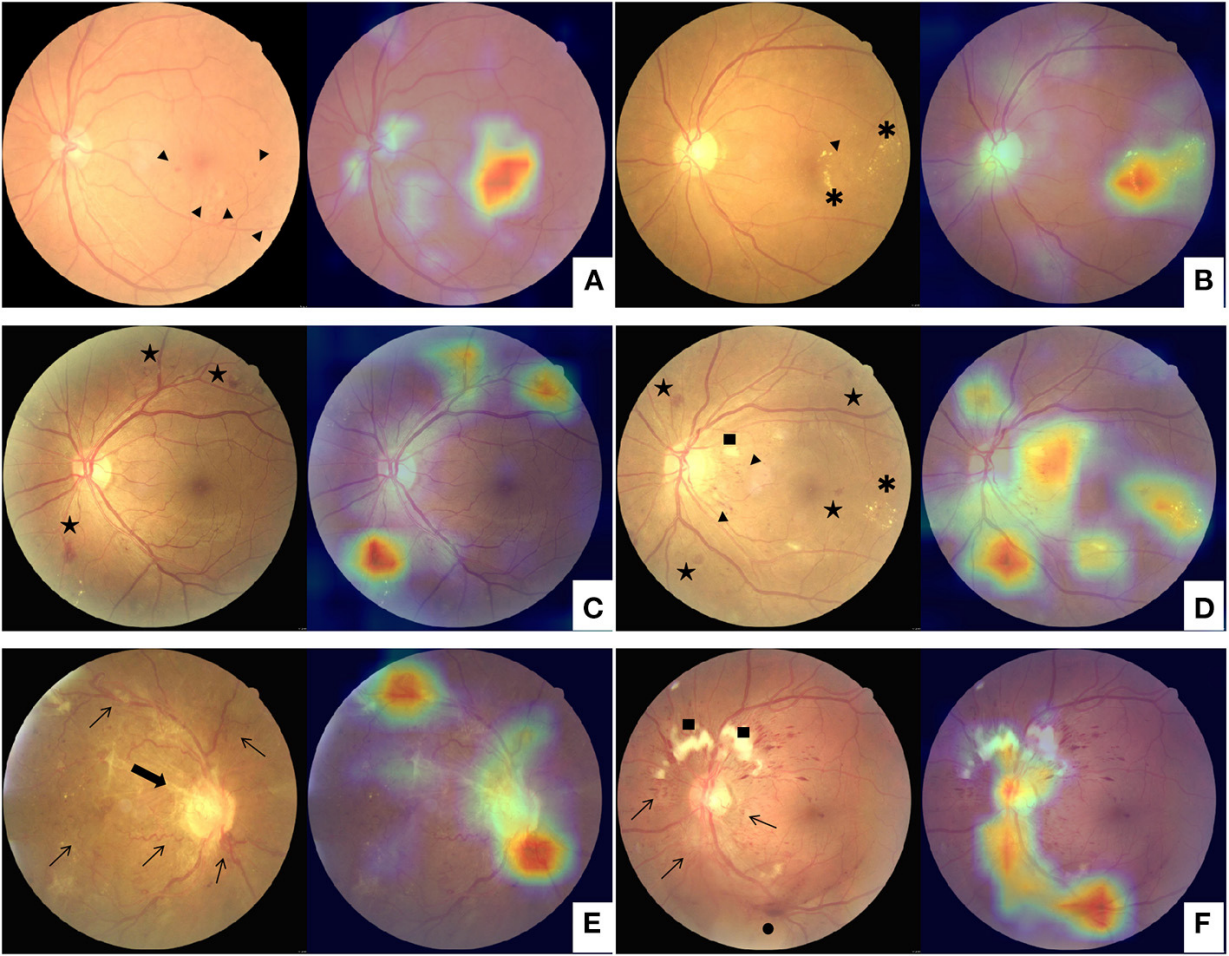
Examples of class activation mapping of lesion on the fundus images with mild diabetic retinopathy **(A,B)**, moderate and severe diabetic retinopathy **(C,D)**, and proliferative diabetic retinopathy **(E,F)** by automated grading system. The augmented image includes the original fundus images (Left) and one highlighted image that indicates the location of the lesions (Right). Arrowhead: microangiomas; Asterisk: hard exudates; Pentagram: intraretinal hemorrhages; Square: cotton-wool spot; Arrowhead: tail; Thin arrows: neovascularization; Thick arrow: fibrovascular proliferation; Black dot: vitreous hemorrhage.

In addition to the DR stage prediction, a heatmap image was also generated by the network model using the Classification Activation Mapping (CAM) technique, similar to highlighting the highly prognostic related regions in the input image (18). The heatmap visualization identified image regions of retinal hemorrhage, exudate, neovascularization, venous beading and looping, etc., which were typical clinical findings associated with DR (Figure 1). Based on the visualization output, the human graderscould substantiate the validity of the deep learning models and promote the clinical adoption of the AI-based automated grading system. Furthermore, the model and parameters were adjusted to point out the site of lesions more precisely to make the algorithm proper for education. The negative images determined by the algorithm would not present any heatmap to avoid confusion. The predicted lesion sites would be highlighted in positive images to guide the participants.

All graders except the two retinal specialists (conducting DR assessment over 20 years) were masked from the annotation results of each other. Three senior ophthalmologists were involved in annotating the training dataset. The reference standard was built up when all the three senior graders draw the same conclusion on the training dataset, and the discordant findings were adjudicated by the two retinal specialists.

### Trainees' evaluations

To mimic the performance of less experienced human graders, two graders who volunteered to participate in the experiment were recruited: a junior ophthalmology trainee in the first year of residency and a medical student who has completed basic medicine courses. After reviewing the traditional lecture of DR before the trial began and being briefed about the annotation protocol, they diagnosed and evaluated 200 images of validation datasets loaded randomly.To evaluate the capability of the training course, 1 month later, the graders were given the extra training course comprising 200 AI-augmented color fundus photos with heatmap images that indicate the location of lesions. After that, they were required to re-annotate the whole validation dataset with the same protocol and made diagnosis.

Then, 120 students in their final year of medical school training who had entered the clinical rotation were recruited from the Medical School at Shandong University to learn diagnosis and grading of DR through this AI-based learning module. After completing the module, participants' evaluations of this system were measured by a 15-item questionnaire rated on five-point Likert-type scales (ranging from “strongly agree” to “strongly disagree”) (Table 1). Using the Questionnaire Star APP (a professional questionnaire survey app in China, easy to edit and distribute survey questionnaires), the questionnaire was devised from the previous studies in other subjects of medical education (19–21). Information of the questionnaire consisted of three parts, which included: basic understanding (three items), domain-based impact evaluation (six items) and respondent's attitude (six items).

**Table 1:** 15-item questionnaire.

**No**.	**Question**
One-choice questions (A, strongly agree; B, agree; C, neutral; D, disagree; E, strongly disagree)	
**Basic understanding**	
1	I think current development of ophthalmic AI is good
2	I regularly encounter AI systems in my clinical practice
3	I regularly encounter AI systems in my training and education
**Domain-based impact evaluation**	
4	AI-based automated grading and training system improved my training and education
5	AI-based automated grading and training system is more effective and motivate
6	AI-based automated grading and training system challenged me to do my best
7	AI-based automated grading and training system promoted the learning of essential concepts or skills
8	AI-based automated grading and training system increased reading of the textbook by the students
9	AI-based automated grading and training system is beneficial to help me to develop critical and creative thinking
**Respondent's attitude**	
10	There is currently sufficient training in AI in my clinical training curriculum
11	More training in AI should be made available for medical students in the education of ophthalmology
12	I will be willing to incorporate AI-based automated grading and training system into my clinical training curriculum
13	I will recommend AI-based automated grading and training system to other students
14	I believed that AI teaching in ophthalmology will replace ophthalmology practice
15	I believed that AI teaching in ophthalmology will replace traditional ophthalmology courses

### Statistical analysis

The accuracy, sensitivity and specificity metrics of the algorithm's outputs and manual grading results were calculated and then compared with the reference standard using StatsModels version 0.6.1 (Python). To evaluate the discriminatory ability of this automated DR grading system, the area under the receiver operating characteristic curve (AUC) was also calculated. The 95% confidence intervals (CIs) were offered meanwhile. After online survey collection, internal reliability of the survey questions was measured by calculating Cronbach's alpha. Descriptive statistics and analysis were carried out using SPSS version 24 (SPSS, Inc., Chicago, IL, USA).

## Results

A comparison of the 4-stage DR diagnosis distribution between the automated grading results and the reference standard on the validation dataset was summarized in Table 2.

**Table 2:** Confusion matrix for adjudicated reference standard and automatic DR grade system output according to modified protocol based on ICDR grading system.

**Reference standard**	**Automated DR grading system**				
	**Non-DR**	**Mild DR**	**Moderate and Severe DR**	**PDR**	**Total**
Non-DR	1435	19	4	4	1462
Mild DR	27	534	59	5	625
Moderate and Severe DR	1	19	554	17	591
PDR	3	1	3	81	88
Total	1466	573	620	107	2766

DR, diabetic retinopathy; ICDR, international clinical diabetic retinopathy; PDR, proliferative diabetic retinopathy.

Table 3 demonstrated that the overall accuracy, sensitivity/specificity and AUC of the grading system for referable DR were 0.965, 0.965/0.966 and 0.980 (95% CI: 0.976–0.984), respectively. The grading system also achieved higher positive predictive value (PPV)/negative predictive value (NPV) of 0.901/0.988, and lower false positive (FP)/false negative (FN) of 0.035/0.035, than that of previous report for referable DR (22). Similar results were demonstrated when we examined other levels of DR according to ICDR grading system (Supplementary Table 1).

**Table 3:** Two graders with/without artificial intelligence assistance verse automatic grading system on referable diabetic retinopathy detection.

	**Automatic grading system**	**Junior resident**		**Medical student**	
		**w/o AI asst**	**With AI asst**	**w/o AI asst**	**With AI asst**
SEN	0.965	0.910	0.972	0.838	0.976
SPE	0.966	0.993	0.994	0.921	0.946
AUC	0.980 (0.976–0.984)	0.952 (0.941–0.962)	0.982 (0.975–0.989)	0.880 (0.864–0.895)	0.961 (0.954–0.969)
ACC	0.965	0.973	0.987	0.901	0.965
PPV	0.901	0.976	0.974	0.775	0.901
FP	0.035	0.007	0.009	0.079	0.035
NPV	0.988	0.971	0.991	0.946	0.988
FN	0.035	0.090	0.028	0.162	0.035

AI, artificial intelligence; SEN, sensitivity; SPE, specificity; AUC, area under curve; ACC, accuracy; PPV, positive predictive palue; FP, false positive; NPV, negative predictive value; FN, false negative; w/o: without, asst, assistance.

Both human graders achieved decent pre-training scores of the grading system for referable DR on the validation dataset (Table 3). However, the score improved in varying degrees when the graders were assisted with the AI system's output. The accuracy of human graders, i.e., junior resident and medical student, was improved from 0.973 and 0.901 to 0.987 and 0.965, respectively. The post-training AUC of the junior resident and medical student for referable DR were 0.982 and 0.961, respectively. Most notably, for the junior resident, the grading sensitivity showed remarkable improvement with AI support (0.910 vs. 0.972). While for the medical student, the improvement was even more pronounced (0.838 vs. 0.976).

As presented in Tables 4, 5, similar results were demonstrated when they grade any levels of DR according to ICDR grading system. In comparing the pre- and post-training scores of different degrees of DR, we identified a significantly higher gained sensitivity of mild DR in the junior resident (0.766 vs. 0.928) and medical student (0.714 vs. 0.838). Moreover, the automated DR grading system increased the graders' sensitivity without reducing the specificity, which was consistent with previous report (23).

**Table 4:** Manual detection of diabetic retinopathy based on ICDR grading system by the junior resident.

				**Junior resident**			
	**w/o AI asst**				**with AI asst**			
	**Non-DR**	**Mild DR**	**Moderate and Severe DR**	**PDR**	**Non-DR**	**Mild DR**	**Moderate and Severe DR**	**PDR**
SEN	0.949	0.766	0.905	0.830	0.967	0.928	0.968	0.909
SPE	0.886	0.945	0.992	0.997	0.969	0.974	0.993	0.996
AUC	0.917 (0.907–0.928)	0.856 (0.838–0.873)	0.949 (0.937–0.961)	0.913 (0.874–0.953)	0.968 (0.961–0.975)	0.951 (0.941–0.962)	0.980 (0.973–0.988)	0.953 (0.923–0.983)
ACC	0.919	0.905	0.974	0.992	0.968	0.964	0.987	0.994
ACC*			0.947				0.978	

AI, artificial intelligence; DR, diabetic retinopathy; ICDR, international clinical diabetic retinopathy; PDR, proliferative diabetic retinopathy; SEN, sensitivity; SPE, specificity; AUC, area under curve; ACC, accuracy; w/o, without, asst, assistance. ACC^*^ represents the overall accuracy for all evaluated images.

**Table 5:** Manual detection of diabetic retinopathy based on ICDR grading system by the medical student.

	**Medical student**							
		**w/o AI asst**			**with AI asst**		
	**Non-DR**	**Mild DR**	**Moderate and Severe DR**	**PDR**	**Non-DR**	**Mild DR**	**Moderate and Severe DR**	**PDR**
SEN	0.900	0.714	0.811	0.636	0.920	0.838	0.954	0.898
SPE	0.925	0.920	0.918	0.993	0.989	0.950	0.949	0.992
AUC	0.913 (0.902–0.923)	0.817 (0.798–0.835)	0.864 (0.847–0.881)	0.814 (0.764–0.865)	0.954 (0.947–0.962)	0.894 (0.879–0.909)	0.952 (0.942–0.961)	0.945 (0.913–0.977)
ACC	0.912	0.873	0.895	0.981	0.952	0.925	0.950	0.989
ACC*		0.915				0.954	

AI, artificial intelligence; DR, diabetic retinopathy; ICDR, international clinical diabetic retinopathy; PDR, proliferative diabetic retinopathy; SEN, sensitivity, SPE, specificity; AUC, area under curve; ACC, accuracy; w/o, without; asst, assistance. ACC^*^ represents the overall accuracy for all evaluated images.

**Figure 2:**
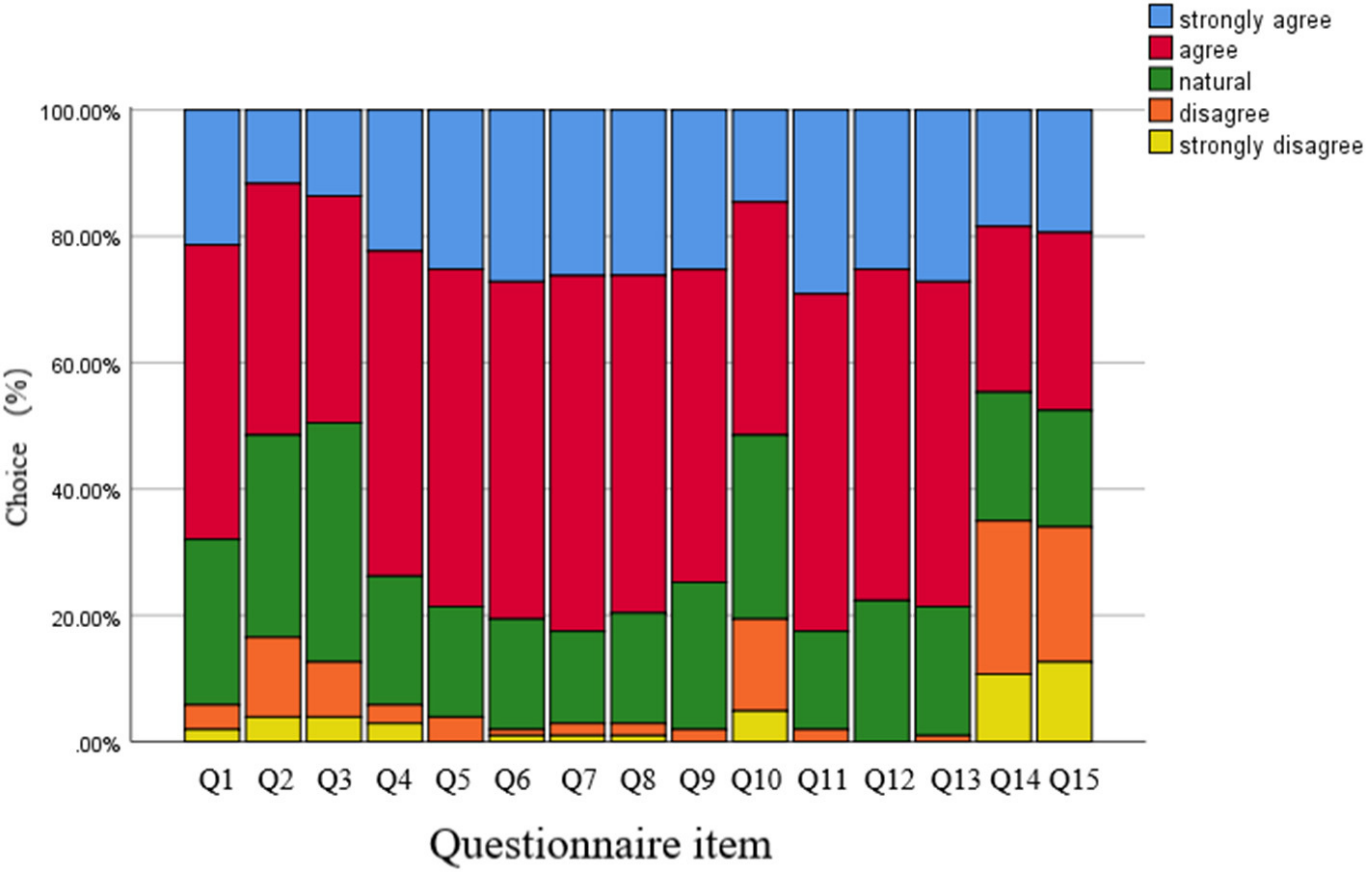
Results of the student evaluation questionnaires regarding the artificial intelligence based grading system. Each survey question used a five-point scale. For details of the questionnaire, see the Table 1.

As shown in Figure 2, of the 120 respondents, 103 students responded to the online survey (response rate 85.83%; 50.49% female). Overall, there was high internal reliability of the survey questions (Cronbach's alpha 0.93). The percentage of respondents who regularly encounter AI systems in their clinical practice and training and education was almost 50%. Over 70% of the trainees agreed that AI respondents were satisfied, helpful and effective. The percentage of respondents who supported more formal AI training was 80 %, while only 14.56% reported sufficient AI training in their current curricula. The AI-based system motivated initiative of trainees, but couldn't replace the traditional ophthalmology practice and courses (18.45%, 19.42%).

## Discussion

In this study, we finetuned an automated DR grading and training system on a real-world diabetic dataset of China and evaluated the diagnostic accuracy as well as its assistance to human graders.

The involved automated grading system achieved high diagnostic accuracy (0.965) and AUC (0.980) for the detection of vision-threatening referral DR on the validation dataset. The PPV of the automated DR grading system achieved 0.901 on referable DR differentiation, which showed high consistency of the grading system with the reference standard. Compared with previous studies (22), high sensitivity (0.965) and NPV (0.988), and more excellent performance on avoiding FN (only 0.035 on grading referable DR) of this system were demonstrated. False positive instances were mainly caused by misclassifying the mild DR as referable DR, while false negative cases were mainly due to the misclassification of referable DR as mild DR. Intra-retinal microvascular abnormalities were identified as the main source of misclassification (24), which was optimized in the training datasets (both laboratory and real-world clinic workflow). False positive cases are mainly due to mild DR being misclassified as referable DR, and false negative cases are mainly due to refeOur automated DR grading system showed great potential as an efficient and low-cost assistant to human graders to detect referral DR patients who need closer follow-up with retinal doctors.

The AI-based DR grading system also revealed its capability to be an effective tool for quantitative assessment of trainees' diagnostic accuracy on fundus images collected from real-world clinics. In conventional DR courses, students are generally taught several foundational knowledge such as fundus anatomy, pathology, grading standards, and representative images of lesions, which are essential for the identification and diagnosis of the disease. Before AI-assisted learning, the two volunteer graders in this study achieved high accuracy as well as high sensitivity and specificity on referable DR and each level of DR independently, which indicated that traditional lectures played a key role in the process of understanding how to diagnose and grade the disease correctly. The integration of theoretical knowledge and clinical practice, however, is the most difficult and crucial aspect yet for medical students. On the other hand, the AI-based DR diagnostic system improved the grading ability of the two trainees, and the medical student with AI assistance even outperformed the junior resident without it. Previous studies have proven that topics that are visually intensive, detail-oriented, and difficult to conceptualize are particularly well-suited for computer-based presentation (25). The grading system provided a large number of fundus images which were collected from the real-world diabetic clinic and generated lesion-emphasized heatmap which can establish linkage between fundamental knowledge and real-life practice. Thermograms generated by AI highlighted the different lesions in real fundus photographs, strengthening students' understanding of pathological characteristics. After grading, the system could give the correct answer immediately, helping students to improve their learning efficiency. The timely monitoring and specific feedback provided by the system allowed students to identify learning goals and knowledge gaps, summarize and analyze their conceptual misunderstandings. Therefore, with training, students can improve the accuracy of their diagnosis by studying a limited number of patients. Our research also revealed that this module significantly considerably improved the students' sensitivity to mild DR detection (e.g., 97% for referable DR, respectively), which was crucial for screening of DR. We thus assumed that this additional course could to some extent compensate for the lack of background knowledge.

Regarding the trainees' classification mistakes, there were two potential causes. First off, due to technological limitations, we were only able to provide a heatmap rather than an arrowhead that points directly to a lesion. In fundus photographs that show multiple lesions at the same time, inexperienced medical students may find it difficult to distinguish between these different lesions. Second, persistent, intentional practice, frequent reinforcement, and expert teacher leadership were required to develop the trainees' ability to classify. Since, repeated practice in a controlled environment is very important in simulation-based education (26), even with more efficient instruments, constant work is still necessary for a successful education. Regarding teacher support, AI can only be used for low-level supervision, so that they cannot completely replace human teachers who can provide additional supervision, intensive training, coaching, and on-going support for students. The teacher's explanation of the clinical analytical thinking processes is critical to the development of students' clinical reasoning ability.

Moreover, the young generation of medical professionals grow up in the era of the internet (27), and there is almost no obstacle for them to use this AI-based system. According to the questionnaire, majority of medical students were more attentive and active during the training process. There is optimism that it will improve their learning of essential concepts or skills and facilitate high diagnostic accuracy with limited learning cases by using this system (28). In common with other surveys, majority of medical students reported their appetite for formal AI training in ophthalmic clinical curricula (29, 30). Although previous research has shown that eLearning is comparable to, and possibly superior to, traditional learning in terms of knowledge, skills, attitudes and satisfaction (31), the current medical school curriculum has not yet fully adapted to these educational needs. Medical students should have sufficient knowledge and experience of artificial intelligence, including its strengths and weaknesses, which is a crucial obligation for future doctors. However, only a small percentage of the population has ever received AI training, and a substantial portion of medical students lack a basic knowledge of these techniques. Although the application of ophthalmic AI goes quickly, AI training of medical trainees in ophthalmology was insufficient. We recommend that the AI-based education system should be integral to the improvement in ophthalmic medical education, especially in diagnosis and grading of DR.

The medical student and the junior resident represent the average diagnosis level of Chinese rural doctors. As we know, regular follow-up with early detection and treatment of vision-threatening DR enables a lower rate of vision loss, making DR no longer the leading cause of blindness among working-age adults in some regions of the world (32). Unfortunately, there are no more than 6,000 specialized doctors in retina diseases in China with uneven distribution around the country (4), screening for DR by ophthalmologists will not be immediately possible. In the longer term, training of the primary care physicians is an effective way to resolve this contradiction. Very little, if any, clinical experience and insufficient training in DR management contribute to lower diagnostic accuracy on DR (33). Thus, ophthalmic education is essential not only for future ophthalmologists but also for non-ophthalmic practitioners in the outpatient clinic. Application of the AI-based training system represents a possible solution to the increasing demand for DR grading education. Advanced technology has enabled learners in resource-limited settings to connect to other individuals, faculty, and even other curricula (34). Compared to traditional education, the automated DR grading and training system would potentially improve rural doctors' ability of DR grading even in limited resource settings.

Beyond the aforementioned key strengths of this study, some limitations must also be considered. First of all, diabetic macular edema (DME) was not involved in this study. This was because we choose optical coherence tomography (OCT) instead of fundus images to determine the presence of DME in the daily clinic, which might make DME underappreciated herein. Secondly, the automated DR grading system might overlook retinal diseases other than DR which might influence the FP rate. The involved images were collected from diabetic clinics, which may excludeother retinal diseases, e.g., age-related macular degeneration myopic maculopathy, retinal vessel occlusion, relatively infrequent or unintentionally (35). Last but not least, the sample size was relatively small, which might affect the validity of the study, the results require to be validated with larger sample size. Given that AI has yet not been widely implemented in clinical practice, there may be legitimate concerns about its instructional use. In order for optimal efficacy, AI-based teaching and learning systems should be rigorously evaluated through expert opinion and multi-institutional studies.

## Conclusion

In summary, the proposed AI-based automated DR grading achieved high diagnostic accuracy for the detection of referral DR and each level of DR according to the modified protocol of ICDR grading system. It can aid the human graders to improve their diagnostic accuracy and sensitivity especially to those lacking didactic training on DR management. Furthermore, it can be used as assistant training system for medical students to experience the real scenarios which makes the traditional lectures properly illustrated. To give the system the essential curriculum knowledge for contextually driven education, further refinement of the system is necessary. Given the explosive recent growth of DM, and the lack of proven models for DR screening, the AI-based DR diagnostic system may be potential for establishment of appropriate primary care system of diabetes and as great importance in aiding medical education.

## Data availability statement

The original contributions presented in the study are included in the article/Supplementary materials, further inquiries can be directed to the corresponding author.

## Ethics statement

The studies involving human participants were reviewed and approved by the Institutional Review Board of Qilu Hospital of Shandong University (QLHSDU). The patients/participants provided their written informed consent to participate in this study.

## Author contributions

QY was responsible for concept and design, supervised the project, guarantors of this work, had full access to all of the data in the study, take responsibility for the integrity of the data, and the accuracy of the data analysis. XQ and HJ wrote the original manuscript draft and were responsible for statistical analysis. XQ mainly takes charge of writing and researching, in the final version of the article, and is tagged as the only first author. All authors contributed to the article and approved the submitted version.

## Funding

Publication of this article was sponsored by the Undergraduate Education and Teaching Reform and Research Project of Shandong University Cheeloo College of Medicine (Grant No. qlyxy-202012) and Education and Teaching Reform Project of Shandong University (Grant No. 2021Y139).

## Conflict of interest

The authors declare that the research was conducted in the absence of any commercial or financial relationships that could be construed as a potential conflict of interest.

## Publisher's note

All claims expressed in this article are solely those of the authors and do not necessarily represent those of their affiliated organizations, or those of the publisher, the editors and the reviewers. Any product that may be evaluated in this article, or claim that may be made by its manufacturer, is not guaranteed or endorsed by the publisher.
