# Supplementary material for: The effectiveness of artificial intelligence-based automated grading and training system in education of manual detection of diabetic retinopathy
Source: Front Public Health. 2022 Nov 7;10:1025271. doi: 10.3389/fpubh.2022.1025271 (PMC9678340; doi:10.3389/fpubh.2022.1025271)
Supplement: Supplementary file 1 [file Table_1.docx]

**Supplementary Table** **1** Performance of artificial intelligence-based automated grading system and manual detection of diabetic retinopathy according to modified protocol based on ICDR grading system

|  | Automatic grading system | | | |
| --- | --- | --- | --- | --- |
|  | No DR | Mild DR | Moderate & Severe DR | PDR |
| SEN | 0.9815 | 0.8544 | 0.9374 | 0.9205 |
| SPE | 0.9762 | 0.9818 | 0.9697 | 0.9903 |
| AUC | 0.9895 (0.9859~0.9931) | 0.9616 (0.9507~0.9725) | 0.9907 (0.9875~0.9939) | 0.9714 (0.9437~0.9991) |
| ACC | 0.979 | 0.953 | 0.9628 | 0.9881 |
| ACC* | 0.9707 | | | |

DR, diabetic retinopathy, ICDR, international clinical diabetic retinopathy, PDR, proliferative diabetic retinopathy, SEN, sensitivity, SPE, specificity, AUC, area under curve, ACC, accuracy. ACC* represents the overall accuracy for all evaluated images.
